# Hangings attended by ambulance clinicians in the North East of England

**DOI:** 10.29045/14784726.2021.12.6.3.49

**Published:** 2021-12-01

**Authors:** Gary Shaw, Lee Thompson, Graham McClelland

**Affiliations:** North East Ambulance Service NHS Foundation Trust ORCID iD: https://orcid.org/0000-0001-5279-1412; North East Ambulance Service NHS Foundation Trust ORCID iD: https://orcid.org/0000-0002-0820-1662; North East Ambulance Service NHS Foundation Trust ORCID iD: https://orcid.org/0000-0002-4502-5821

**Keywords:** emergency medical services, hanging, suicide

## Abstract

**Introduction:**

Suicide rates have risen in England over the last decade and hanging, a highly lethal method of suicide, has been the most common method. Previous work in this area identified a lack of literature discussing emergency medical services (EMS) attendance at hangings. This article aims to describe hangings attended by EMS in the North East of England in order to inform future work in this area.

**Methods:**

A retrospective service evaluation was conducted using existing data from a comprehensive pre-hospital trauma audit database to describe patients with hanging documented in their records who were attended by ambulance clinicians between 1 December 2018 and 31 November 2020.

**Results:**

Hanging was recorded in 604 incidents. Most cases (n = 579/604) involved adults (aged 18 years or older) with a median age of 35 years (IQR 27–45 years), who were male (n = 410/579, 71%). Just over half (n = 341/579, 59%) of adult hangings resulted in cardiac arrest and of these, 10% (n = 33/341) were resuscitated and survived to hospital admission. Threatened and non-fatal hangings appear to have increased dramatically in the latter half of 2020. Previous suicide attempts and mental health issues were frequently reported across this population.

**Conclusion:**

Hangings are a method of suicide which frequently result in a cardiac arrest. In the North East of England the ambulance service attends approximately one hanging per day and one fatal hanging every two days. When fatal hangings were resuscitated, pre-hospital outcomes were similar to other causes of cardiac arrest, highlighting that despite the traumatic nature of these cases resuscitation is not futile. In order to better understand this patient group and improve care, pre-hospital data need to be linked to data from other services such as mental health services and acute hospitals.

## Introduction

According to the World Health Organization (WHO), suicide is responsible for 1.4% of all deaths worldwide, and for every adult that dies by suicide there are over 20 attempts (WHO, 2020). The annual report from the Office for National Statistics (ONS) (ONS, 2020) shows that suicide rates in England and Wales have increased over the last decade. The North East had the third highest rate of male suicides in 2019, with a rate of 19.1 deaths per 100,000 males against an average of 16.9. Hanging is a highly lethal method of suicide, and hanging, strangulation and suffocation has been the most common method of suicide used by males and females in the UK since 2013 (ONS, 2020). A recent scoping review highlighted the limited literature on hangings attended by emergency medical services (EMS) and identified areas for further study, including the changing frequency, the demographics of the patient population and the impact of these cases on clinicians and bystanders (Shaw et al., 2021).

Two recent articles have provided some early evidence relating to the impact that attending suicides, such as hangings, has on EMS clinicians, including the lasting emotional impact and the desire for better training, as well as a recognition of the impact on bystanders and family members (Nelson et al., 2020; Rothes et al., 2020).

The aim of this service evaluation is to report the incidence of hangings attended by North East Ambulance Service NHS Foundation Trust (NEAS) clinicians, describe the characteristics of the patients and inform further discussion and research in this area with the ultimate aim of improving the care delivered to these patients.

## Methods

A retrospective service evaluation was conducted using existing data from a comprehensive pre-hospital trauma audit database to describe patients with hanging documented in their records who were attended by NEAS clinicians over a two-year period.

### Setting

NEAS is the regional ambulance provider for around 2.7 million people in North East England, covering Northumberland, Tyne and Wear, County Durham, Darlington and Teesside. NEAS employs around 1200 ambulance clinicians (paramedics and other clinical roles), who work out of 56 stations across the region (NEAS, 2020).

### Data collection, extraction and analysis

All electronic patient care records (ePCRs) describing hangings were extracted from the daily pre-hospital trauma audit by specialist trauma paramedics (GS and LT) between 1 December 2018 and 31 November 2020. The ePCRs were identified based on the following inclusion criteria:

Inclusion:

- Any mention of hanging in diagnosis code or free text; and
- Any age.

The following data, including 58 individual fields, were selected by the authors as being relevant to hangings based on a previous scoping review (Shaw et al., 2021). Data were manually extracted from the ePCRs by the authors and sorted into the following domains:

- Demographics;
- Call timings;
- Clinical observations;
- Past medical history;
- Staff in attendance;
- Interventions;
- Patient destination and/or outcome; and
- Narrative information relevant to hanging.

Patients included in this study were stratified into: out-of-hospital cardiac arrest (OHCA) due to the hanging; or not in cardiac arrest (non-OHCA). The non-OHCA population were subdivided into those who had hanged themselves and those who had threatened to hang themselves.

All data are presented in a descriptive fashion along with the number of cases where data were available. Summary statistics are presented using median, inter-quartile range (IQR) and range. Narrative information was extracted from the free-text sections of the ePCRs and summarised.

## Results

This evaluation identified 604 ePCRs where hanging was documented between 1 December 2018 and 31 November 2020. Hangings involving children (defined as a patient under the age of 18 years) accounted for 25/604 (4%) of cases, with the remainder being adults.

### Children

The majority of children were teenagers (median age 16 years, IQR 15–17), with a female bias (64% female). There were six children aged below 15 years in the sample. Just under half of cases involving children were OHCA (n = 11/25, 44%) of which three were transported to hospital due to return of spontaneous circulation (ROSC) being achieved.

The majority of child non-OHCA cases (n = 14/25, 56%) were GCS 15/15 (n = 11/13, 85%), half were found by family or carers (n = 7/14, 50%) and most had recorded mental health problems (n = 8/13, 62%). Non-OHCA cases tended to happen at or around the weekend (Friday to Monday, n = 10/14, 71%) in the evenings (17:00–00:00, n = 10/13, 77%).

### Adults

**Figure 1:**
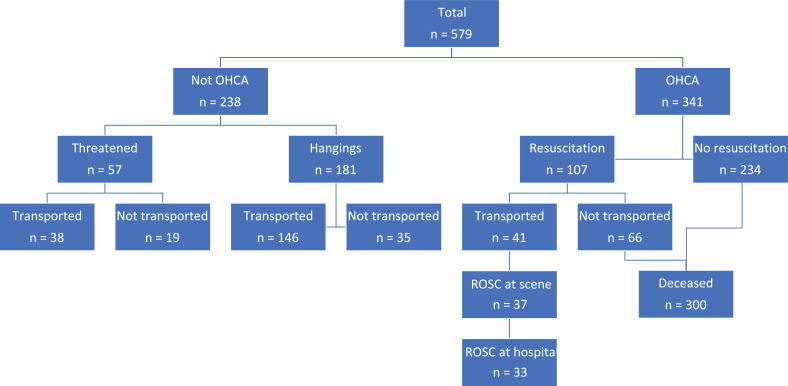
Figure 1. Adult patients with documented hanging.

All data from this point refer to the 579 adult cases (Figure 1). The median age (n = 572) was 35 years old (IQR 27–45, range 18–90) and the majority were male (n = 410/579, 71%).

Within the study, there were four patients who were included multiple times, two male and two female, all under 40 years old, all with documented mental health issues, and between them they accounted for 13 non-OHCA calls.

**Figure 2:**
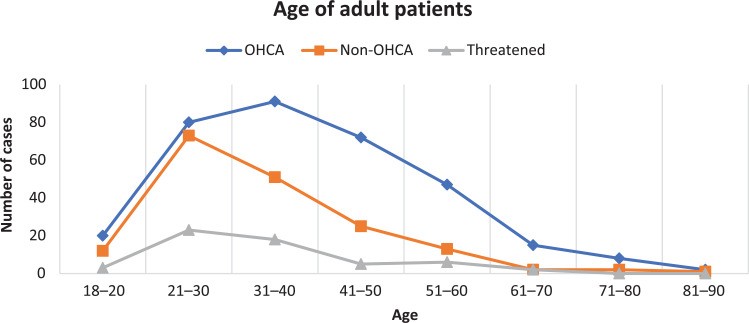
Figure 2. Age distribution of adult hangings.

The demographics and characteristics of included adult patients are reported in Table 1. The table highlights the three groups of OHCA, non-OHCA and threatened hangings and their relationship with recorded previous mental health issues, alcohol/drug use and whether C-spine trauma was present and/or managed. The pattern of ages is shown in Figure 2.

**Table 1.** Demographics and characteristics of adult patients.

	OHCA	Non-OHCA	Threatened
**Patients**	341	181	57
**Age (median, IQR)**	38 (28–49)	32 (25–40)	33 (24–40)
**Sex (male)**	78%	60%	63%
**Transported by NEAS**	15 (11%)*	81%	67%
**Mental health history**	96 (28%)	126 (70%)	47 (82%)
**Previous suicide attempt**	17 (5%)	61 (34%)	28 (49%)
**C-spine trauma/deformity**	33 (10%)	5 (3%)	0
**C-spine collar applied**	13 (4%)	22 (12%)	5 (9%)
**Alcohol reported**	29 (9%)	60 (33%)	16 (28%)
**Drugs reported**	13 (4%)	20 (11%)	5 (9%)
**Alcohol and drugs reported**	10 (3%)	17 (9%)	3 (5%)
**Day of week for incident**			
**Saturday**	45 (13%)	36 (20%)	13 (23%)
**Sunday**	60 (18%)	29 (16%)	10 (18%)
**Monday**	63 (18%)	28 (15%)	15 (26%)
**Tuesday**	42 (12%)	29 (16%)	3 (5%)
**Wednesday**	49 (14%)	22 (12%)	9 (16%)
**Thursday**	48 (14%)	23 (13%)	8 (14%)
**Friday**	45 (13%)	22 (12%)	5 (9%)

* 15% OHCA transported but 11% transported with ROSC or continuing resuscitation.

The most common days for OHCA hangings to be reported were Monday and Sunday. The most common days for non-OHCA hangings to be reported were Tuesday, Saturday and Sunday. The most common days for threatened hangings to be reported were Monday, Saturday and Sunday (see Table 1).

**Figure 3:**
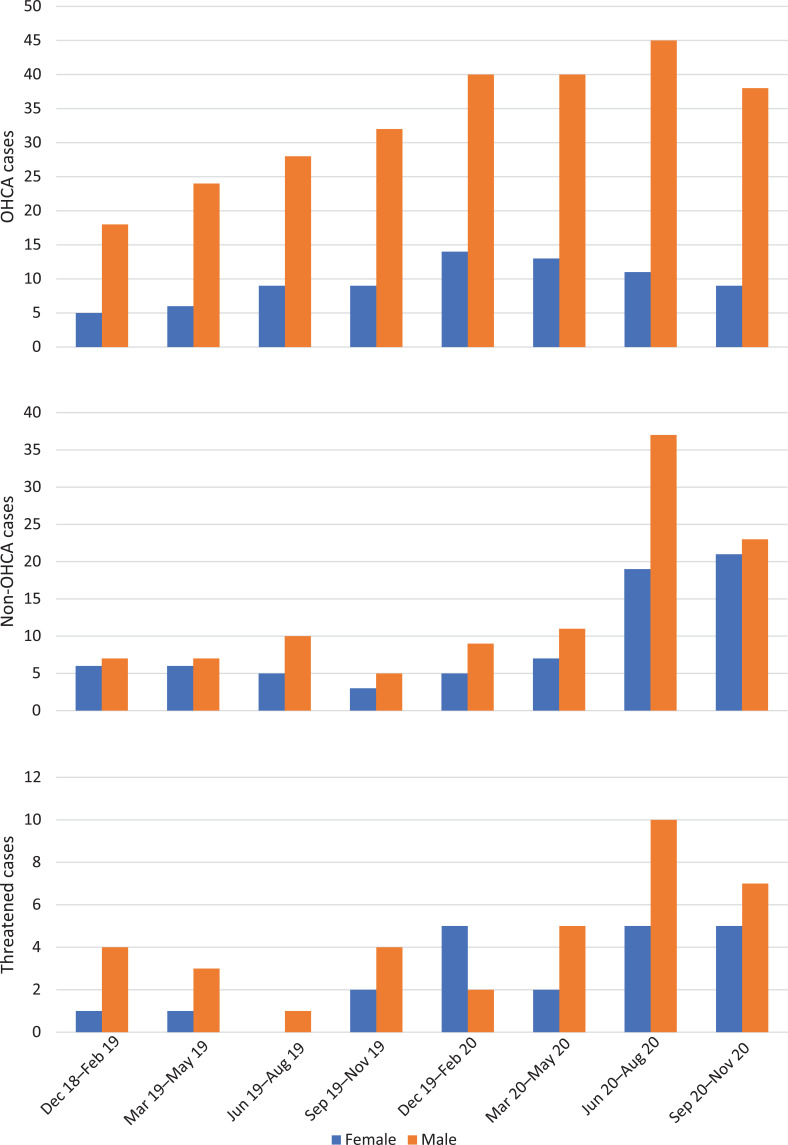
Figure 3. OHCA, non-OHCAS and threatened cases by sex and quarter year.

The cases included in the study are reported by sex and in three-month quarters in Figure 3.

### Location

There were no obvious differences in patterns between sexes with regards to locations. Home was the most frequent location for people to be found, with the hallway and bedroom the most common rooms. Outdoors was the next most common location for people to be found, with other notable locations including prison/police cells (see Table 2).

**Table 2.** Location, ligature type, suspension, ‘found by’ and suicide note demographics of hangings.

Location	n (%)
**Home**	412/579 (71)
Hallway	96/412 (23)
Bedroom	58/412 (14)
**Outdoors**	105/579 (18)
Wooded area	51/105 (49)
Public space	18/105 (17)
Park	14/105 (13)
**Prison/police cell**	18/579 (3)
**Hospital**	12/579 (2)
**Ligature type and suspension**	
**Type of ligature recorded**	394/579 (68)
Clothing	131/394 (33)
Cable/cord/wire	91/394 (23)
Rope	84/394 (21)
**Ligature marks documented**	183/579 (32)
OHCA ligature marks	101/183 (55)
OHCA ROSC ligature marks	18/101 (18)
Non-OHCA ligature marks	70/183 (38)
Threatened ligature marks	12/183 (7)
**Suspended off ground documented**	55/579 (10)
Off ground with ligature marks documented	26/55 (47)
**Found by and time to discovery**	
**‘Found by’ documented**	519/579
Family/partner	225/519 (43)
Police/emergency services	119/519 (23)
Public	49/519 (9)
**Time to discovery documented**	274/579 (47)
Threatened witnessed	21/22 (95)
Non-OHCA witnessed	44/73 (60)
**Median time to discovery**	**Minutes (IQR)**
OHCA	360 (60–1440)
OHCA + ROSC (n = 24)	30 (15–60)
OHCA + No-ROSC (n = 155)	480 (180–1440)
**Suicide notes/multimedia**	
Suicide note/multimedia documented	109/579
OHCA	68/109
Non-OHCA	35/109
Threatened	6/109
Notes/letters	41/109 (38)
Text message	40/109 (37)
Phone call	31/109 (28)

### Ligature and suspension

In 394/579 (68%) cases, the material or item used as a ligature was recorded. The most common ligatures were clothing, cable/cord/wire and rope. Ligature marks were documented on 183 (32%) patients.

The patient was documented as being ‘off the ground’ in 55 (9%) cases, with trees (n = 20) the most frequent suspension point when being off the ground was documented (see Table 2).

### Who found the patient and when they were last seen

The person who found the patient was documented in 519 (90%) cases. There were 274 (47%) patients who had a time window for when they were last seen documented. The majority (n = 21/22) of threatened hangings with times recorded were witnessed and the only one not witnessed was seen <15 minutes before the incident. Most (n = 44/73) non-OHCA hangings with time recorded were also witnessed and in those that were not witnessed the median last seen time was 14 minutes (IQR 5–60).

The median last seen time in OHCA patients (n = 179) was 6 hours (IQR 1–24). However, the median time for OHCA patients who achieved ROSC (n = 24, including only one >60 minutes) was 30 minutes (IQR 15–60) compared to 480 minutes (IQR 180–1440) for OHCA patients who did not achieve ROSC (n = 155) (see Table 2).

### Suicide notes and other media

Suicide notes or other methods of contacting people were documented in 109 (19%) cases, with a median age of 36 years (IQR 26–50; 74% male). The most common methods were notes or letters, followed by texts and phone calls. These methods were not mutually exclusive, and some people used multiple methods including social media (see Table 2).

### Combative, agitated or aggressive

Twenty-eight patients were recorded as combative, agitated or aggressive (one OHCA, two threatened, 25 non-OHCA; median age 30 (IQR 25–38)), including 20 males and eight females. NEAS front line crews were supported by specialist paramedics or helicopter emergency medical services (HEMS) at six of these cases, of which four received some combination of midazolam (n = 4), diazepam (n = 1) and RSI (n = 1). Half of the 28 combative, agitated or aggressive patients (n = 14) had alcohol consumption documented and four had drug use documented. Most (n = 19, 68%) of these patients had a history of mental health problems.

### Adult OHCA hangings

There were 341 adult hangings resulting in OHCA (see Table 3). NEAS attempted to resuscitate 107 (31%) patients, 84 (79%) of whom had received bystander CPR. Return of spontaneous circulation (ROSC) was achieved at scene in 39 patients (36% of resuscitations), 36 of whom received bystander CPR. ROSC was sustained to hospital in 33 patients (26% of resuscitations), 31 of which received bystander CPR. Bystanders provided CPR in 103 (30%) cases overall. The presenting electrocardiograph (ECG) rhythm was documented for 135 (40%) of OHCA patients, as described in Table 4.

**Table 3.** Adult OHCA hangings: Resuscitation, return of spontaneous circulation, resources and interventions.

	n (%)
**OHCA (adult)**	341
Bystander or NEAS resuscitation	126/341 (37)
NEAS resuscitation	107/341 (31)
Bystander CPR	103/341 (30)
Combined bystander and NEAS CPR	84/341 (25)
Return of Spontaneous Circulation (ROSC) on scene	39/126 (36)
ROSC in those receiving bystander CPR	36/103 (35)
ROSC sustained to hospital	33/126 (26)
**Resources on scene**	
NEAS paramedic	338/341 (99)
Specialist trauma/emergency care paramedics	48/341 (14)
Helicopter Emergency Medical Service (HEMS)	14/341(4)
Hazardous Area Response Team (HART)	10/341 (3)
Mechanical chest compressions	11/126 (10)
Airway management	85/107 (79)
Supraglottic (main)	68/107 (64)
Endotracheal tube (main)	12/107 (11)
Oropharyngeal (main)	4/107 (4)
Nasopharyngeal (main)	1/107 (<1)
Semi-rigid cervical collar	13/341 (4)
Recognition of life extinct (ROLE)	303/341 (89)

**Table 4.** Presenting ECG rhythm of OHCA patients.

Presenting rhythm	Number of patients (%)	Median age (IQR)	Resuscitated by NEAS (%)	ROSC at scene (%)	ROSC at hospital (%)
Asystole	121/341 (35)	36 (28–47)	53/121 (43)	15 (12)	13 (11)
VF/VT	2/341 (1)	31	2/2 (100)	1/2 (50)	1/2 (50)
PEA	12/341 (4)	31 (28–43)	12/12 (100)	8/12 (75)	6/12 (50)
Not recorded	206/341 (60)	39 (30–50)	40/206 (19)	15/206 (7)	13/206 (6)

Mechanical chest compression, delivered by the LUCAS device brought by either HEMS or trauma/specialist paramedics, was used in 11/126 cases (10%) of resuscitation attempts. Seven of these patients were conveyed to hospital, with four achieving ROSC at scene and three sustaining it to hospital.

The majority (n = 85/107, 79%) of patients where NEAS attempted resuscitation had some form of airway management recorded. A supraglottic airway was used in 68 (64%) cases, an endotracheal tube was used in 12 (11%) cases, an oropharyngeal airway was used in four (4%) cases and one patient had a nasopharyngeal airway placed.

Semi-rigid cervical collars were applied to 13 (4%) OHCA patients. All OHCA patients who were collared were transported with ROSC at scene and none of these patients had recorded cervical spine trauma/deformity.

Out of the 341 adult OHCA hangings, 303 (89%) were recognised as deceased by NEAS (recognition of life extinct (ROLE)). The majority of these were recognised as deceased at scene and only a small number (n = 13, 4%) of these patients were moved from the location where they were found. Patients who were moved were mostly outdoors in public spaces.

Although only two patients had shockable rhythms recorded as the presenting rhythm, 11 patients had documented defibrillation shocks. The median number of shocks was one (IQR 1–3), with the maximum shocks delivered being five. Seven patients who received shocks achieved ROSC at scene, six of whom sustained ROSC to hospital.

## Discussion

The aim of this service evaluation was to describe hangings attended by NEAS clinicians in order to report the incidence of hangings seen by NEAS, describe the characteristics of the patients and inform further discussion and research in this area with the ultimate aim of improving the care delivered to these patients.

### Description of results

Between December 2018 and November 2020, NEAS attended 604 hanging cases. This equates to an incidence of six hangings per week, with one hanging resulting in OHCA roughly every two days. The population described was largely young males, which reflects national trends (ONS, 2020) and data from a neighbouring ambulance service (Hayden et al., 2021). Paediatric hangings were thankfully rare and reflected previously reported patterns (Shaw et al., 2021).

As seen in Figure 3, the number of OHCA hangings was rising steadily and then plateaued early in 2020; however, the number of non-OHCA and threatened hangings rose dramatically in mid-2020, possibly influenced by the COVID-19 pandemic and lockdown (McClelland et al., 2020). The ratio of males to females was largely consistent over time, with males making up 71% of the population overall; however, as shown in Table 1 and Figure 2, OHCA hangings were younger and more likely to be male than non-OHCA or threatened hangings.

The majority of non-OHCA and threatened patients had known mental health problems. The lower figure in OHCA patients may reflect a lack of available medical history or differences in documentation. A small number (n = 4) of patients, all with documented mental health problems, were included in this evaluation multiple times, perhaps illustrating that their underlying mental health needs were not being met.

Cardiac arrests (OHCAs) made up the largest sub-group in this study. NEAS resuscitated a third of OHCAs, most of whom had bystander CPR (Alqahtani et al., 2020), and the ROSC to hospital rate was 10% in the OHCA group overall. The ROSC to hospital rate in patients who were resuscitated was 31%, which is the same as the average national ROSC to hospital rate for all OHCAs between April 2018 and March 2020 (NHS England, 2021), showing that OHCAs related to hangings are not hopeless cases.

Eleven patients were defibrillated, even though only two presented in a shockable rhythm, and 55% of these sustained a ROSC to hospital. This suggests that non-shockable rhythms were converted to shockable rhythms by clinician interventions and that the presence of a shockable rhythm is a positive indicator in these patients.

Specialist resources were dispatched to 99 cases, most of which (n = 64) were OHCA. The NEAS specialist emergency care paramedics launched in November 2019 so were not available for the full period described. The small number of cases attended by clinicians with varying levels of additional skills or expertise precludes drawing any conclusions from these data about their impact on hangings. There were a small number of patients (n = 28) who were described as agitated or combative in these data. These patients, as well as any OHCA patients who achieve a ROSC who may be agitated due to hypoxia, may benefit from the attendance of clinicians with the knowledge, training and ability to sedate or anaesthetise them safely in the pre-hospital setting.

### Strengths and limitations

This article represents a detailed description of a large sample of hangings attended by EMS across a regional ambulance service. The inclusion of OHCA patients, non-OHCA patients and threatened patients gives a broad view of the patient population, which may be difficult to capture from other sources of data. The patients declared deceased at scene will be a difficult population to collect data on without ambulance involvement.

Limitations of this study include the use of routinely collected data which reflect what the clinician completing the documentation considered pertinent at the time. Many fields of interest, such as alcohol, ligature marks and material used, are not mandatory so there may be missing data. Clinicians documenting OHCA cases may complete the ePCR with different priorities in mind than when completing non-OHCA records. Inclusion of a specific hanging section with mandatory data fields in the ePCR would improve the quality of the data.

### Generalisability

The North East has historically had a higher rate of suicide than average (ONS, 2020) so there may be local factors limiting generalisability. In addition, around half of the time described was during the COVID-19 pandemic which may limit generalisability to non-pandemic times.

The North West Ambulance Service published data on suicide deaths attended by their clinicians between 1 January and 28 June 2019 (Hayden et al., 2021). This article described 124 patients who were 71% male with an average age of 44 and where hanging was responsible for 92% of all deaths, which is in line with national data (ONS, 2020).

### Controversies

Cervical spinal trauma/deformity was documented in 10% of OHCA hangings and 3% of non-OHCA hangings, none of whom had cervical collar application documented. The terminology used here may be one source of this potential disconnect, as cervical spinal trauma may have been documented due to the mechanism of injury rather than any physical evidence (Thompson et al., 2019). The inconsistent use of cervical collars (7% overall), particularly in the OHCA population, needs further exploration. The fact that collars were only used in OHCA patients with ROSC who were transported suggests that these were applied to stabilise the patient during extrication and movement as opposed to due to concern for a cervical spinal injury. The changing attitude towards cervical spinal collar use and the de-emphasis in trauma guidelines may contribute to this variation in use.

The use of the internet and social media to seek help or post suicide notes is a relatively recent development which raises challenges and opportunities to intervene and help people at risk (Robinson et al., 2016). In this study, 4% of people who left any form of note/communication used social media, but this figure may be under-reported as ambulance clinicians would not normally enquire about patients’ social media use.

### Implications for practice and research

The authors believe that the data presented highlight issues for practice and opportunities for further research, including:

- The emotional impact on clinicians’ mental health from attending hangings needs to be considered, especially for those in specialist roles who may be exposed to these otherwise rare instances on a regular basis;
- Management of the airway in a hanging patient and the risk/benefit ratio of intubation needs to be considered with the removal of this skill from many paramedics;
- The risk/benefit ratio of cervical spinal collars for a hanging patient and clear guidance on if and when these should be used;
- How to deliver advanced interventions such as sedation or rapid sequence intubation to agitated or combative patients to enable safe management;
- The benefits of linking ambulance data to other sources (e.g. primary care, mental health, coroners) to explore the events before and after ambulance involvement.

## Conclusion

Hangings are a method of suicide which frequently results in a cardiac arrest. The North East ambulance service attends approximately one hanging per day and one fatal hanging every two days. Threatened and non-fatal hangings appear to have increased dramatically in the latter half of 2020. When fatal hangings were resuscitated, pre-hospital outcomes were similar to other causes of cardiac arrest, highlighting that despite the traumatic nature of these cases resuscitation is not futile. In order to better understand this patient group and improve care, pre-hospital data need to be linked to data from other services such as mental health services and acute hospitals.

## Acknowledgements

The authors wish to thank the many people who have encouraged them to pursue this piece of work and those who will build on this work going forward.

## Author contributions

All authors conceived the study. GS and LT collected the data. GM analysed the data and drafted the manuscript. All authors read and approved the final manuscript. GM acts as the guarantor for this article.

## Conflict of interest

GM is the Editor-in-Chief of the editorial board of the *BPJ*.

## Ethics

Not required.

## Funding

None.
